# The Use of Humor in Serious Mental Illness: A Review

**DOI:** 10.1093/ecam/nep106

**Published:** 2011-01-03

**Authors:** Marc Gelkopf

**Affiliations:** ^1^Department of Community Mental Health, Faculty of Social Welfare and Health Sciences, University of Haifa, Haifa, Israel; ^2^Lev Hasharon Mental Health Center, PO Box 90000, Netanya 42100, Israel

## Abstract

There is now a relatively good understanding of the broad range of direct and indirect effects of humor and laughter on perceptions, attitudes, judgments and emotions, which can potentially benefit the physical and psychological state. This article presents a review and discussion of the use of humor and laughter in treating people with serious mental illness, distinguishing between clinical papers on individual and group psychotherapy, and empirical research reports describing humor and laughter interventions. In spite of the exponential growth of the field over the last 30 years, I conclude that empirical studies are still lacking, the studies that do exist have major methodological shortcomings, and the field is in dire need of further investigation.

From the laughter of god were born the seven gods that govern the world … When he had laughed, the light appeared [*⋯*]. He laughed for the second time: everything was water. At the third laugh appeared Heres; at the fourth, creation; at the fifth, destiny, at the sixth, time itself. Then, before the seventh laugh, he took a great breath, but having laughed so much, he cried and from the tears sprang the soul.

       Anonymous, third century [1]

## 1. Introduction

Laughter and humor have existed in all societies throughout the ages, and play an important role in many mythologies worldwide [2]. Many ancient philosophers, such as Aristotle and Plato, and later, Descartes, Hobbes, Locke, Kant and Darwin, as well as modern philosophers such as Bergson, Jankelevich and Alvin Toffler, have considered humor or laughter to be a central phenomenon in the lives of people and societies [3].

The place of humor in mythologies and the attention it has received by philosophers attests to its importance for the delicate balance between human well-being, divinity and society. But the use of humor is not limited to storytelling and theorizing. The well-known proverb: “a merry heart doeth good like a medicine” (Proverbs 17:22) has been used through the ages, and records show that physicians have been advocating the potentially curative aspects of humor for hundreds of years.

Although the “science of humor” is a relatively young field, research from the last 30 years has suggested the mechanisms through which humor may positively impact health [4]. Humor seems to have the potential to effectuate pain relief [5], strengthen immune function [6], improve positive emotions [7], moderate stress [8–11], dissociate from distress [11–13] and improve interpersonal processes [14, 15].

In the wake of the above-mentioned studies, we have seen the growing application of humor and laughter interventions with child [16] and adult [17] medical patients, as well as its use in psychotherapy frameworks [18, 19].

Probably due to what seems to be the therapeutic potential inherent in humor and laughter, the noteworthy sense of humor observed in many of the “super-therapists,” such as Ellis, Perls, Erickson, Satir, Rogers or Whitaker and the growing use of humor in general hospitals [16, 20, 21], we have seen an increase in the use of humor in individual and group psychotherapy and in a number of humor and laughter interventions within psychiatric institutions and with individuals with “serious mental illness” (SMI).

SMI is usually used to refer to severe and long-lasting mental disorders such as major depression (MDD), schizophrenia (SZ), bipolar disorder (BD), obsessive compulsive disorder (OCD), panic disorder, post-traumatic stress disorder (PTSD) and borderline personality disorder (BPD). SMIs are conditions that disrupt a person's motivation, thought processes, emotions, mood, interpersonal relationships and behaviors.

Challenges for these individuals can be very encompassing and range from regaining meaning in life, self-esteem, coping with strong anxiety, depressive and suicidal thoughts, coping with traumatic experiences, coping with strong hostility and sexual drives interpersonal and intrapersonal conflicts; including shame and guilt, loss of the ability to enjoy life, social alienation and internalized stigma as well as institutionalization [22].

These conditions often necessitate significant intervention. But treatment is notably difficult, and interventions include mainly medication, various therapies and psychosocial education interventions aimed at rehabilitation. Therapeutic challenges include the establishment of a positive working alliance to optimize treatment adherence, working through strong resistance, instilling hope and a positive outlook on life, and by working through the strengths of the client, encouraging him to regain interpersonal and vocational skills.

Modern practices and orientations in rehabilitation do not advocate per se the eradication of symptomatology, but focus upon community integration, improvement in quality of life through reduction and control of symptoms as well as client empowerment. Nevertheless many clients regularly feel helpless to cope with symptoms, and find themselves hospitalized.

**Figure 1:**
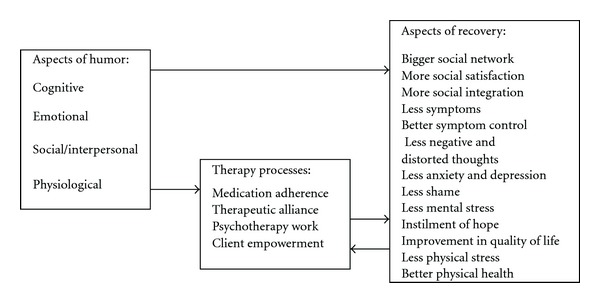
Potential ways in which humor can contribute to recovery.

Humor in this frame of reference could be used as an adjunct to conventional treatment with the goal of helping clients cope with symptoms, improving rehabilitation through its emotional, cognitive, social and physiological impact as well as reinforcing and facilitating therapy and client empowerment (Figure 1).

In the following paragraphs, I shall present a review of the literature that presents the theoretical and clinical background describing the potential benefits of the use of humor in individual and group therapy for SMI followed by a review of the empirical studies on the use of therapeutic humor with different SMI populations.

## 2. Potential Benefits of Humor and Laughter in the Treatment of SMI

### 2.1. Use of Humor and Laughter in Individual Psychotherapy

The use of humor in psychotherapy with patients with chronic and serious mental illness has been widely described. Advocates of its use have come from all major psychotherapy orientations, including existentialists [23, 24], dynamic therapists [25], behaviorists [26] cognitivists [19], paradox-oriented therapists [27, 28], family therapists [29], Gestalt therapists [30], provocative therapists [31] and others, and it has even been presented as an important aspect of supportive therapy for caregivers of people suffering from SMI [32]. The techniques used in the many approaches vary widely and have been described elsewhere [33].

Based upon the aforementioned clinical and research work, I have made the classification of the potential contributions of humor to individual therapy for people with SMI as presented in Table 1.

### 2.2. Humor in Group Therapy

In his book, *The Expression of the Emotions in Man and Animals*, Darwin [49] speculated that the evolutionary basis of laughter had its function as a social expression of happiness, and that this rendered a cohesive survival advantage to the group. More recently, the understanding of the evolutionary development and social survival value of humor has been well described [50]. In support of this theory, laughter has been found to occur in social contexts over 95% of the time [51]. In addition to regulating conversation [52], laughter enhances social relations by producing pleasure in others through simple contagious processes [53] and by rewarding others' actions, thus encouraging ongoing social activities [54]. Studies of the role of social laughter demonstrate its pro-social benefits, for example, by increasing group cohesiveness [55].

Therefore, although the use of humor in group therapy makes the same kind of contribution as in individual therapy, it has its own distinctive advantages. This is based primarily on the extent of its impact (e.g., making a group laugh is different than making an individual laugh), the social contagion effect (e.g., more opportunities for catharsis) and the opportunities facilitated by social exchanges in group format (e.g., working on social skills). The use of laughter and humor in groups may be especially relevant to the SMI population because one of the major problems of this population is the lack of social skills accompanied with high levels of social alienation. In addition, psychiatric settings may be especially suited to the optimal use of social laughter because much if not most of the therapeutic activities in such settings take place in group format. Based on the descriptive work of some researchers [25, 56–59], I suggest the classification of the contribution of humor and laughter to group psychotherapy in psychiatric settings, as summarized in Table 2.

## 3. Empirical Studies on Humor-Oriented Interventions with SMI

### 3.1. Non-Tailored Interventions

Although interventions “tailored” to the individual or group would seem to be the most effective way of using humor, some approaches suggest that the laughter itself is enough to promote at least short-term well being. Indeed, laboratory studies have suggested increases in positive mood in subjects following forced non-humorous laughter [62]. “Yoga laughter” has been used as an intervention mode in a number of health settings [63], and today, there are hundreds of “laughter classes” worldwide, including psychiatric settings, although none has been investigated scientifically [64].

However, two studies assessed the use of non-tailored laughter on chronic schizophrenic patients in a psychiatric hospital [15, 65, 66]. In both studies, humorous movies were shown twice daily (five times weekly) to 17 and 15 SZ patients, respectively, in wards, over a 3-month period, and “regular” movies (including 15% humorous) were shown to control wards. These were assessed both before and after, using well-established self- and clinician-rated questionnaires. These studies suggest that the use of this kind of intervention may reduce psychiatric symptomatology, anxiety, depression, verbal hostility and aggression, anger, and improve social support and social competence. On the other hand, this intervention did not improve physical health-related measures, nor did it improve the therapeutic relationship with the therapist. Interestingly, the first study [67] showed no relationship between any improvement between any of the measures, and a patients' ability to enjoy humor, supporting the suggestion of Falkenberg et al. [68] that such interventions may be beneficial for many of the patients, regardless of their ability to enjoy humor. The first study, where the staff had the opportunity to watch the movies with the patients also showed an improved relationship between staff and patients [15], and based upon qualitative ward observation, an improvement in ward atmosphere. However, the second study showed that the impact of this intervention was not mainly due to its social aspect, because in the second study improvements could be observed on both psychiatric symptomatology, depression and anxiety, as well as social competence without the staff watching the movies, and without any improvement in the relation with the staff, nor any change in ward atmosphere.

One major criticism of these studies is that they did not assess “laughing” itself thus it is possible that some other aspect of comedy viewing, such as social expectation or positive emotions, may be responsible for the effect. Furthermore, no “dose effect” could be observed in patients who watched more versus those who watched fewer movies, suggesting it may be a general “positive atmosphere” affecting the wards, and not specifically the laughter itself.

### 3.2. Humor Groups in the Psychiatric Setting

A number of interventions have been set up with the declared aim of using humor as a therapeutic tool to improve the quality of life of mental health clients. The best known is probably *Stand Up for Mental Health* [69], which uses stand-up comedy, including training and public performances to enhance self-competence, sense of control and self-worth, as well as reducing self-stigma, and uses the performances to educate the public about stigma. Although these and others are noteworthy, only a handful of interventions have been empirically assessed.

Witztum et al. [70] performed an empirical 6-month intervention in a psychiatric ward with 12 schizophrenic patients. Based upon the paradoxical *ad absurdum* principle, described by Frankl [23], Titze [27] and Whitaker [28], with the aim of creating paradoxical scenarios which would obviate judgment errors and the irrationality of behavior patterns on the part of the patients, all 12 patients were offered humorous renderings and interpretations of their most prominent complaints, which had been prepared in advance to debunk the alleged symptoms. This 3-month humor intervention was scheduled after a 3-month version of Ellis' Rational Emotive Therapy (RET). The humor approach was more efficient in reducing psychopathological symptoms than the RET, as assessed by the Brief Psychiatric Rating Scale (BPRS) for the same patients.

Minden [71] described 4 years' work in an open group that provided patients with a mirthful place for respite and shared laughter. The intervention was held as an “open group” in the psychiatric wards, where each patient was offered to participate in six to eight 1-h sessions. Each weekly session included an introduction, which was conducted in a playful manner and set a jovial tone for group interaction. The “call for jokes” served as a springboard for more spontaneous humor, provided an incentive to prepare ahead of time, and gave more introverted participants a concrete focus. Next, a “humorous activity” engaged members in a variety of games, songs, dances, skits or relay races that emphasized cooperation. This activity was followed by a discussion that encouraged members to share concerns and plan for future sessions and was a safeguard against humor's divisive or destructive potential. Finally, there was an “enlightenment” component, which often took the form of an instant replay of some funny group occurrence and ended the session on an upbeat note. After each session, a debriefing was held in which the students and the instructor critiqued the group process and developed strategies for improvement. A total of 66 sessions were held, involving 129 patients. Interviews conducted with 13 patients revealed several themes suggestive of the group's therapeutic value. The participants viewed the group as a place in which was a sense of connection developed, communication improved and social skills were learned. They also learned to regulate thoughts and feelings, attain new perspectives, reduce stress and enhance coping, find respite and relaxation, and laugh with others at oneself. A similar intervention was also described by the same author [72] within a forensic psychiatric setting. This was performed in a naturalistic setting without a control group or systematic data gathering.

Walter et al. [40] compared the impact of pharmacological medication and humor group therapy (*n* = 20) to standard medication treatment alone (*n* = 20) in elderly patients with late-onset depression and Alzheimer's disease. The Humor group received 1 h therapy once every 2 weeks. During each the moderator acted as a stimulant for humor, smiling and laughter using verbal techniques. After an initial phase, the moderator told or read humorous stories or suggestive funny anecdotes. The aim was to trigger the patients' reactions, observations or comments by way of personal associations. Where appropriate, the moderator intervened with provocative or slapstick humour. Furthermore, happy biographical episodes and memories were also addressed later on with the aim of creating shared laughing or smiling. The focus of the humor therapy was the accentuation of an exhilaration milieu in the group and the encouragement of everyone's sense of humor.

In this pilot study, it has been shown that although the humor intervention reduced depression symptoms, improved mood, daily living activities and quality of life for a group of 10 depressive patients, it did so as much as the standard medication treatment group. In addition, none of the interventions had any effect on any of the measures for the Alzheimer group. This study suffered from many shortcomings, mainly a very heterogenic population regarding cognitive ability, and that participants participated in 2–12 sessions thus rendering comparisons impossible as well as what seems to be an unregulated post questionnaire administration.

Roller and Lankester [73] described how an open-ended group for clinically depressed out-patients worked, using both humor and paradoxical intent during 100–120 1 h sessions. This intervention was found to be successful for most of the 80 group participants who succeeded in overcoming depression symptoms, as determined by achieving some success in either the discontinuation of medication, obtaining a job or establishing a positive relationship or friendship. In this study no control group was used, and assessment was done on the basis of files only.

### 3.3. Medical Clowns

Historically and culturally, clowns have been associated with the well-being of society and the healing arts. Since the late 1980's, several clowning approaches have entered general hospitals with the basic aim of providing empowerment, a supportive relationship and the opportunity for play, especially for children, but also for adults. The techniques used vary widely, not only between approaches (e.g., clown doctors, therapeutic clowns, therapeutic play) but also depend upon the clown's own abilities.

Although medical clowning is now widespread and in use in thousands of general hospitals worldwide, very little research has studied its impact both in pediatric [16] and cancer [17] ward settings. A literature search, as well as informal networking, found only two studies assessing the impact of such an intervention on psychiatric wards.

In a 6-week intervention pilot project involving 27 older patients with SMI, with affective and psychotic diagnoses, and 22 staff members, Wild et al. [74], found a positive change of attitudes regarding the helpfulness of clowns for improving emotional states in patients. The intervention included six visits of two clowns in the psychiatric ward where they both gave a “personal” show (which averaged 8 min) for each patient as well as a 30-min group intervention for 21 of the 27 patients. Six patients refused any contact with the clowns. The patients were questioned using a structured nine-item questionnaire developed for that study, regarding their acceptance of the clowns and the personal benefit they experienced. In general most of the 21 patients appreciated the clowns, and their appreciation grew over time. Nevertheless no control group was used, the questionnaire only queried the appreciation of clowns, and not the impact it had on well being, and the questioning of patients happened at different time points for each patient making it difficult to assess any real impact.

A study by Higueras et al. [75] described the implementation of a bi-weekly clown intervention during two 3-month periods, in a 29-patient closed SMI psychiatric ward, to assess its impact on disruptive behaviors ranging from aggression towards self and staff and attempts to escape the ward. Each session began with warm-up exercises (marching in different directions in time to a set rhythm, stretching and dancing), followed by group activities led by the clowns, and consisting of games, psychomotor expression exercises, activities based on imaginary situations (imitation in front of a mirror, charades, playing with an imaginary ball, visits to an imaginary planet with zero gravity, games based on invisibility, games based on curiosity). Humor was an element in all activities, which took place in a setting of controlled tolerance. At the end of the session, quieter games were usually played to lower the level of excitement. Results suggest an overall reduction of disruptive behavior on all measures assessed. Blind assessments were made based on standard reports of disruptive behaviors during the intervention period (*n* = 101), and comparisons were made with the period prior to the intervention (*n* = 83). There was a significant reduction in the number of disruptive behaviors during the experimental period. A closer look at the results showed that fewer people attempted to escape the ward; there was less agitation, less aggression towards staff, less self-injury, less fighting and less non-cooperation in the ward.

## 4. Discussion

The therapeutic use of humor has grown exponentially over the last two decades. An abundance of professional and non-professional articles and books have been written on the subject, interventions have been developed and many websites are actively promoting this therapeutic modality. The Association for Applied and Therapeutic Humor (AATH) includes psychotherapists, psychiatrists, counselors, teachers, nurses and other health professionals, many of who actively promote the use of humor in psychiatric settings. A major conclusion of previous reviews on the general effects of humor [4, 6, 18] is that humor has a broad range of effects on perceptions, attitudes, judgments and emotions, which may mediate directly or indirectly to benefit the physical and psychological state.

Although a plethora of studies have described its theoretical underpinnings and its potential use in psychotherapy, empirical studies regarding the potential therapeutic use of humor are scant, especially those assessing its impact on SMI. The studies that have been published have significant methodological flaws; they either lack control groups [70, 75], use non-standardized assessment tools with non-standardized measurement periods [71, 75] or very small samples [65, 66]. Finally, most studies do not have an adequate “emotion-evoking” control stimulus for distinguishing between the effects uniquely attributable to humor and those attributable to positive emotions in general.

Humor or laughter is an easy-to-use, inexpensive [66], natural therapeutic modality that could be used within different therapeutic settings, with a multi-professional staff whose impact could, at the least, temporarily alleviate some of the daily distress experienced by the seriously mentally ill. It is, therefore, surprising that it has not been widely applied and researched in psychiatric settings, especially since this population is most in need of cost-efficient means to improve quality of life.

The first reason behind the relative lack of development of humor-related therapeutic intervention in this population may be the history of professional socialization in psychiatry, where the focus on emotional distancing, conformity and hierarchy does not encourage the “risk taking” involved in humorous encounters with clients [76]. Another reason may be rooted in the fact that schizophrenic and populations with SMI have traditionally been considered as impaired in their ability to enjoy humor, although unjustifiably so [77]. They have also been perceived as more vulnerable to the anxiety-evoking aspects of humor, thus disliking humor in general [69, 78] as well as therapeutic interventions incorporating humor [79]. Another reason may originate in the fact that humor is not considered “mainstream,” has a “new-age” unscientific connotation, and may find difficulty receiving financial support for either an intervention or a scientific study. Thus, most humor interventions are applied on a small scale, in “amateurish” ways, and without any scientific assessment.

A further reason may lie in the emotional potential of humor to negatively affect a person. Indeed, many therapists are conscious of the dangers that may accompany the inappropriate use of humor with SMI patients [36, 37], as humor (like all therapy) must be used with skill and sensitivity. For example, in the Stand-Up for Mental Health intervention, it is important to ensure that patients' comedy routines do not become a form of “self-defeating” humor, and this requires implementation by a skilled clinician (and not just a comedian).

A final reason may originate in Western European history of the Middle Ages, when the unholy trinity—the devil, folly and laughter—were to be burned at the stake [2]. Foucault [80] observed, in his history of mental illness, that in the middle ages, the laugh of the madman was the laughter of death. It is possible that the shrill sounds of the witches' laughs still echo within our scientific minds.

Therapeutic humor is, therefore, a unique field in that it is characterized by a discrepancy between clinical practice and scientific research. On the one hand, a plethora of therapeutic approaches has been developed, such as the use of medical clowns, stand-up, paradoxical humor-oriented approaches, humor training and yoga laughter, and many leading and innovative therapists have openly advocated and made use of it and institutionalized it; but on the other hand, only a handful of studies have been published assessing its potential for the seriously mentally ill, a population that is potentially in dire need of life-uplifting experiences.

The present review thus suggests developing empirical research projects to assess the potential use of these modalities with mental health clients, and in institutional settings.

## Figures and Tables

**Table 1:** Potential contributions of humor to individual therapy for people with SMI.

*Diagnostic*: In MDD, OCD as well as schizoaffective depressive symptomatology, strong anxiety, aggressive and sexual impulses shame and guilt may lead to high levels of tension. Given the right therapeutic context this can be expressed through spontaneous laughter or humor which can then be investigated [29]. Patients' jokes can also be considered as a projective tool to assess conflicts [34], and laughter can also be seen as a welcomed and desirable index of the process of therapeutic change itself [30, 35].
*Emotional*: Strong anxiety are a major aspect of many of the disorders classified under SMI. On the other hand certain disorders such as SZ and PTSD are at times characterized by emotional numbness. Laughter can both reduce excessive anxiety and facilitate the expression of emotions [36, 37] such as feelings of hostility [38] that would otherwise become self-defeating. Laughter can also be a mind-relaxing tool, helping to reach emotional content that the patient is neurotically or psychotically protecting, or as a phase in initiating systematic desensitization [26].
*Cognitive*: Distorted cognitions and obsessive rumination are some of the features of many SMI's. Humor can foster self-observation by initiating the reorganization of attitudes (e.g., in regard to specific subjects such as sex, ridicule, or the debunking of catastrophe scripts), and by temporarily suspending taboos and distancing oneself from obsessive thoughts, humor can offer a sense of proportion [33] as well as promote different perspectives towards problems [19]. Humor can also facilitate a pleasurable and hedonistic approach to problems, in stark contrast to depressive or suicidal thinking [33, 39, 40].
*Somatic*: SMI patients suffer from important physical and mental stress. As a natural tension reducer, humor can be used to relieve somatic stress and facilitate therapeutic processes [41]. A number of authors have described the importance of a physiological rapport between therapist and patient [42, 43]. This is especially relevant for SMI patients for whom the establishment of good therapeutic rapport is a major predictor of successful rehabilitation (ref). In a study assessing skin conductance measures of therapists and patients in videotaped psychotherapy settings, Marci et al. [44] has suggested empirically that this rapport is strengthened in the presence of laughter.
*Potential space*: Especially relevant for BPD, humor can promote the use of a potential space of play, where themes can be explored and shared in a non-defensive way [45].
*Dynamic processes of personality*: Humor and laughter can release rigid defenses, promoting communication with unconscious processes, widening the repertory of available coping options and strengthening the ego [46].
*Therapeutic relationship*: Humor in the therapeutic relationship may help deepen the therapeutic alliance, as the use of humor can strengthen the feeling of acceptance, enhancing empathy and a sense of belonging [33]. Therapists can show their humanness and break down barriers that often exist within the therapeutic context—especially within psychiatric institutions [37]. The therapist's spontaneous laughter can improve the patient's trust in the therapist and therapeutic process [47].
*Therapist-related processes*: Outside of the therapeutic context, humor can help the staff in the psychiatric institution deal with frustrating sessions, the processes of institutionalization, and difficult-to-treat chronic patients that may affect burnout [48].

**Table 2:** Potential contributions of humor to group therapy for people with SMI.

*Diagnostic*: The way individuals use humor in group situations can, in certain cases, be an indicator of inadequate social coping means, or can be explored as a positive coping skill that can be generalized to other social situations. Analyzing the timing of laughter may also help understand certain dynamic group processes.
*Emotional*: Due to the social contagion effect of laughter, the potential of emotional catharsis in groups is great. Furthermore, using humor as an outlet for hostility and fear in a group situation may enhance the acceptance of these feelings and the pleasure of making people laugh may boost their expression.
The contagion effect of laughter in groups can lead to both loud and strong laughter and in some instances to “fou rire” or uncontrolled positive laughter, effecting both physical and emotional catharsis and relaxation.
As one major problem for patients with SMI is the deficit in the ability for emotional self-regulation, group humor may be a good setting in which to learn this, due to positive up-regulation of emotions within the group. Indeed, a recent study has shown that humor can serve as a potent cue for emotional up-regulation [59].
*Therapist-related aspects*: The therapist, as a model figure, displays behaviors, including humor, that the patient may imitate [60], and adopt as a coping mode.
*Cognitive*: As in individual therapy, humor within a group context may enable and facilitate the development of a sense of proportion, and may help overcome exaggerated seriousness that often serves as a defense against ambiguity. The presentation of one's life in a humoristic manner may often help patients accept certain difficult situations in a more existential way, accepting life's absurdities and quandaries.
*Social*: Patients' use of humor may strengthen interpersonal skills, social confidence and reduce social phobia very often present in SMI's. In gender- or ethnic-specific groups within psychiatric contexts, humor can be used to strengthen the gender or ethnic identity of the participants [58] and as such foster community integration.
*Group-related aspects*: The use of incongruity and surprise by means of humor may stimulate the group, evoke curiosity, overcome defenses and provide a cue for remembering and internalizing insights more readily than verbal interpretations. Humor in a group context can help the self-disclosure process, thereby contributing to emotional catharsis and strengthening group belonging. It can promote a sense of intimacy, attachment and friendliness in the group, improving cohesion and morale. This aspect is paramount in institutional settings and with patients with SMI, who are often socially alienated.
*Institutional aspects*: Group humor may be especially beneficial in that it sets aside institutional rules to facilitate dialogue between clients and professionals [61]. If humor is used in large groups or in regular ward meetings, its impact may generalize to the larger therapeutic setting of the ward or the clinic.
